# Clinical Relevance of Right Atrial Functional Response to Treatment in Pulmonary Arterial Hypertension

**DOI:** 10.3389/fcvm.2021.775039

**Published:** 2021-12-07

**Authors:** Manuel J. Richter, Daniel Zedler, Dominik Berliner, Philipp Douschan, Henning Gall, Hossein A. Ghofrani, Lucas Kimmig, Nils Kremer, Karen M. Olsson, Bruno Brita da Rocha, Stephan Rosenkranz, Werner Seeger, Athiththan Yogeswaran, Zvonimir Rako, Khodr Tello

**Affiliations:** ^1^Department of Internal Medicine, Justus-Liebig-University Giessen, Universities of Giessen and Marburg Lung Center (UGMLC), Member of the German Center for Lung Research (DZL), Giessen, Germany; ^2^Department of Cardiology, Hannover Medical School, Hannover, Germany; ^3^Division of Pulmonology, Department of Internal Medicine and Ludwig Boltzmann Institute for Lung Vascular Research, Medical University of Graz, Graz, Austria; ^4^Department of Pneumology, Kerckhoff Heart, Rheuma and Thoracic Center, Bad Nauheim, Germany; ^5^Department of Medicine, Imperial College London, London, United Kingdom; ^6^Department of Respiratory Medicine, German Center for Lung Research Biomedical Research in Endstage and Obstructive Lung Disease Hannover (DZL/BREATH), Hannover Medical School, Hannover, Germany; ^7^Klinik III für Innere Medizin and Cologne Cardiovascular Research Center (CCRC), Herzzentrum der Universität zu Köln, Köln, Germany

**Keywords:** pulmonary hypertension, echocardiography, speckle tracking, outcome, right atrium

## Abstract

**Background:** Right atrial (RA) function has emerged as an important determinant of outcome in pulmonary arterial hypertension (PAH). However, studies exploring RA function after initiation of specific pulmonary vascular treatment and its association with outcome in patients with incident PAH are lacking.

**Methods:** RA peak longitudinal strain (PLS), passive strain (PS), and peak active contraction strain (PACS) were retrospectively assessed in 56 treatment-naïve patients with PAH at baseline and during follow-up after initiation of specific monotherapy or combination therapy. Patients were grouped according to their individual RA functional response to treatment, based on change from baseline (Δ): worsened (first Δ-tertile), stable (second Δ-tertile), and improved (third Δ-tertile). The Spearman's rho correlation and linear regression analysis were used to determine associations. Time to clinical worsening (defined as deterioration of functional class or 6-min walking distance, disease-related hospital admission, or death) was measured from the follow-up assessment. The association of RA functional treatment response with time to clinical worsening was assessed using the Kaplan–Meier and the Cox regression analyses.

**Results:** Median (interquartile range) time to echocardiographic follow-up was 11 (9–12) months. Of the 56 patients, 37 patients (66%) received specific dual or triple combination therapy. Δ RA PLS during follow-up was significantly associated with changes in key hemodynamic and echocardiographic parameters. The change of pulmonary vascular resistance, right ventricular (RV) end-systolic area, and global longitudinal strain were independently associated with Δ RA PLS. The median time to clinical worsening after echocardiographic follow-up was 6 (2–14) months [17 events (30%)]. In the multivariate Cox regression analysis, worsening of RA PLS was significantly associated with clinical deterioration (hazard ratio: 4.87; 95% CI: 1.26–18.76; *p* = 0.022). Patients with worsened RA PLS had a significantly poorer prognosis than those with stable or improved RA PLS (log-rank *p* = 0.012). By contrast, PS and PACS did not yield significant prognostic information.

**Conclusion:** Treatment-naïve patients with PAH may show different RA functional response patterns to PAH therapy. These functional patterns are significantly associated with clinically relevant outcome measures. Improvements of RA function are driven by reductions of afterload, RV remodeling, and RV dysfunction.

## Introduction

Pulmonary arterial hypertension (PAH) is a severe multifactorial disease characterized by increased total pulmonary resistance with subsequent right ventricular (RV) pressure overload (1). Increased RV afterload results in adaptive and maladaptive RV remodeling (hypertrophy and dilatation, respectively), eventually leading to RV failure (2). In addition to the right ventricle, remodeling of the right atrium has come into focus in pulmonary hypertension (PH) in the recent years. Alterations of right atrial (RA) function are relevant prognostic markers of adverse outcomes (3, 4). RA function is characterized by three phases: a reservoir phase during atrial filling when the tricuspid valve is closed, a conduit phase during passive emptying of the right atrium into the right ventricle when the tricuspid valve is open, and an active “contractile” phase during atrial systole (contraction) (5). Initially, chronic RV pressure overload causes an increase in RA contractility and RA dilation due to elevated RV diastolic pressure and tricuspid regurgitation (6). RV remodeling leads to impaired RA function, which results in worsening of reservoir (4), conduit (7), and contractile functions (8). The interaction of the right atrium and right ventricle may play a crucial role in PH. A loss of that interaction in the sense of RA-RV “uncoupling” results in alterations of RA function to a failing reservoir phase and an impaired conduit component that are inevitably associated with a reduction of cardiac output and RV filling (6). Most recently, it was shown that longitudinal assessment of RA function after treatment initiation may serve as an additional predictive marker in children with PH (9). However, the clinical relevance of RA functional response to specific vasoactive treatment in adult patients with PAH is currently unknown. Therefore, we aimed to longitudinally assess and characterize RA function in treatment-naïve adult patients with PAH.

## Materials and Methods

### Study Design and Patients

Data from consecutive, treatment-naïve adult patients referred to our PH clinic between December 2017 and April 2020 and enrolled in the prospectively recruiting the Giessen PH Registry (10) were retrospectively analyzed. The diagnosis of PAH was made by the multidisciplinary PH board at the University Hospital Giessen according to the updated recommendations (11). Patients with pacemakers (*n* = 1) or atrial fibrillation or atrial flutter (*n* = 2) at the time of evaluation were excluded. All the patients received individual targeted PAH therapy based on current guidelines and best standard of care (12). Prior to treatment initiation, patients underwent baseline evaluation. Median time between baseline right heart catheterization and echocardiography was 16.5 (2–46.3) days. Invasive pulmonary hemodynamics and pulmonary arterial capacitance (PAC) were measured as previously defined (13). All the participants gave a written informed consent for the enrollment into the Giessen PH Registry. The investigation conforms to the Declaration of Helsinki and was approved by the Ethics Committee of the Faculty of Medicine at the University of Giessen (approval #266/11).

### Echocardiography

All the measurements were performed as recommended by current echocardiographic guidelines (14, 15) and obtained using a Vivid E9 device (GE Healthcare, Wauwatosa, Wisconsin, USA). Tricuspid annular plane systolic excursion (TAPSE) and RV fractional area change (FAC) were quantified. RA area was measured at end-systole. RA pressure (RAP) was estimated by evaluation of inferior vena cava diameters (expiratory and inspiratory) and percent collapse during inspiration. Pulmonary arterial systolic pressure (PASP) was calculated as the transtricuspid gradient + RAP (16). Tricuspid valve regurgitation was graded as mild, moderate, or severe as recommended (17). RV global longitudinal strain was measured as previously described (18).

Echocardiographic images were analyzed by an independent investigator who was not directly involved in the image acquisition and who was blinded to the clinical data. Measurements were made using EchoPac software (version 201, GE Healthcare, Wauwatosa, Wisconsin, USA).

**Figure 1:**
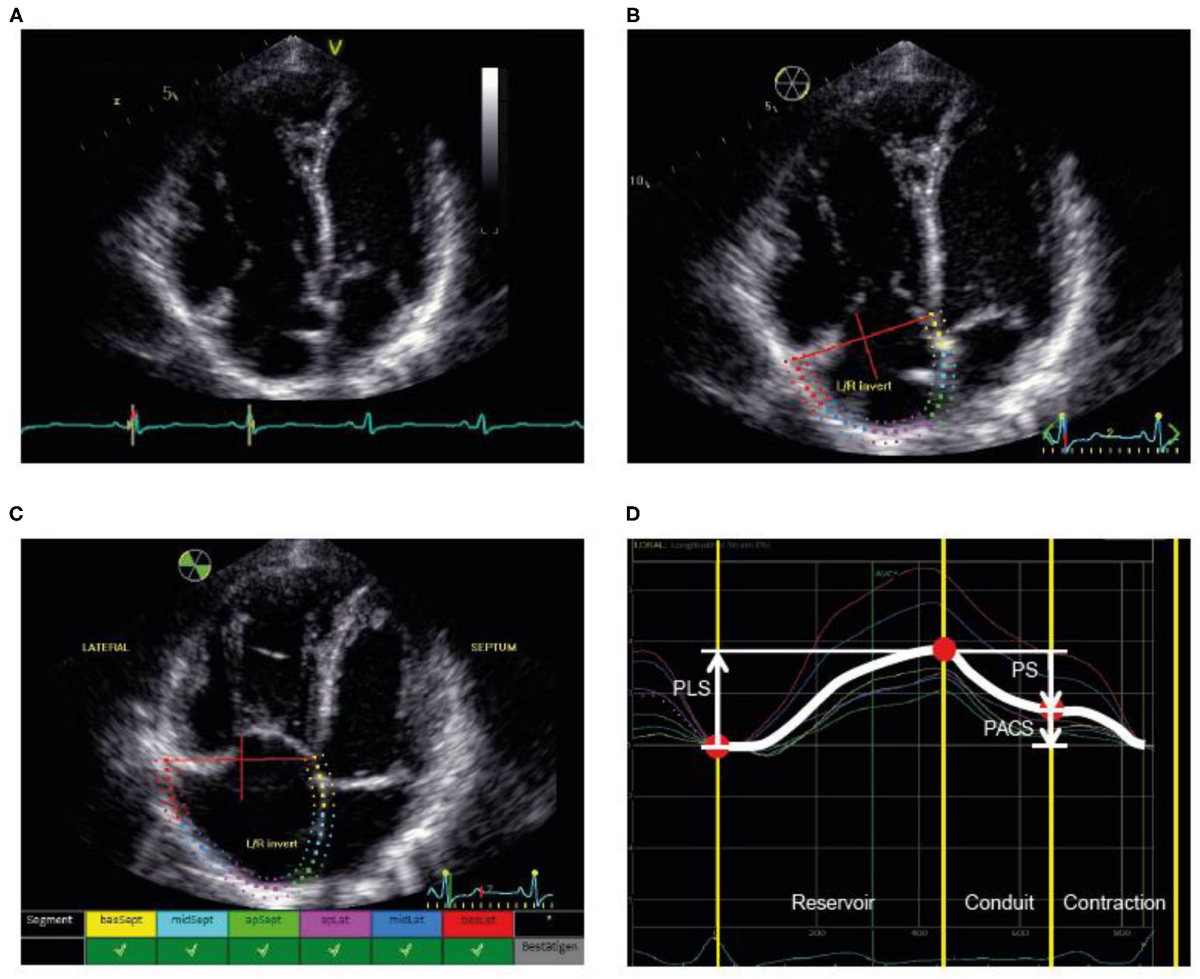
Illustration of the assessment of RA strain. **(A)** First, the RV-focused apical four-chamber view was used with selection of the cardiac cycle and adjustment of the electrocardiogram (to R-wave). **(B)** Second, the RA endocardial border was traced as the region of interest, covering the RA lateral wall, roof, and septal wall. **(C)** Third, processing provided an overview wherever speckle tracking was feasible for the selected regions. **(D)** Fourth, the different phases were identified and the strain values determined. PACS, peak active contraction strain; PLS, peak longitudinal strain; PS, passive strain; RA, right atrial; RV, right ventricular.

Tracing of the right atrium was performed as shown in Figure 1 according to the current recommendations (7, 19, 20). Using a right ventricle-focused apical four-chamber view, the region of interest was manually placed on the RA endocardial border. After automatic tracing of the six segments, every segment was manually adjusted to the thickness of the RA wall. The zero reference was set at the R wave and all the strains were positive. RA peak longitudinal strain (PLS) and peak active contraction strain (PACS) were assessed as measures of the reservoir phase and contraction phase, respectively. RA passive strain (PS), indicative of conduit function, was calculated from the difference between RA peak longitudinal and active strain as shown in Figure 1. Intra- and interobserver variability for PLS were assessed in a random subset (20%) of the cohort.

### Outcome

Clinical worsening was assessed after follow-up echocardiography and was defined as any of the following: reduction in exercise capacity (−15% compared with the baseline 6-min walk test), worsening in the WHO functional class, clinical deterioration requiring hospital admission (need for new PAH therapies or intravenous diuretics), or death (21). Follow-up was assessed until July 2021.

### Statistical Analysis

The Kolmogorov–Smirnov test was used for assessment of normal distribution. The Pearson's chi-squared test, related-samples Wilcoxon signed-rank test, the paired samples *t*-test, the independent samples Kruskal–Wallis test, or the one-way ANOVA was used to analyze differences between groups, as appropriate. The Spearman's rank correlation was used to measure association between variables. Inter- and intraobserver variability were assessed using intraclass correlation coefficients and coefficient of variation.

A backward (based on likelihood ratio) multivariate linear regression model was built to determine the parameter most strongly related to the change of RA function. Variable selection was limited to three variables to avoid overfitting and was based on clinical relevance. Model 1 included the absolute change of mean pulmonary arterial pressure (mPAP), PAC, and pulmonary vascular resistance (PVR). Model 2 included the absolute change of TAPSE/PASP, FAC, and RV end-systolic area. Model 3 incorporated the absolute change of TAPSE/PASP, FAC, and RV global longitudinal strain. Multicollinearity was assessed using the variance inflation factor.

The uni- and multivariate Cox proportional hazards models were built to assess the relationship between RA function and the clinical outcome, with RA function included either as a continuous variable or as a categorical variable based on tertiles (with tertile III, “improved,” set as the reference category). Owing to the limited number of events, adjusted covariates were limited to age and sex. For further evaluation, the Kaplan–Meier analyses with log-rank tests were used, with all the events or censoring times measured from the date of follow-up echocardiography.

For all the analyses, *p* < 0.05 was considered as statistically significant.

The SPSS version 26.0 and 27.0 (IBM, Armonk, New York, USA) and GraphPad Prism version 8.4.3 (GraphPad Software, San Diego, USA) were used for statistical analyses.

## Results

### Patients

The majority of the patients with incident PAH presented with an advanced WHO functional class (Table 1). Baseline pulmonary hemodynamics demonstrated a precapillary pattern of PH with substantially elevated pulmonary pressures and resistance. As shown in Table 2, patients presented with substantial RV and RA enlargement, depressed RV systolic function measured by FAC, and impaired RA function compared with values previously reported in healthy controls (19). Baseline PLS and PACS were associated with the severity of tricuspid regurgitation. We observed significantly higher PLS and PACS values in patients with mild-to-moderate tricuspid regurgitation compared with patients with severe regurgitation (Supplementary Figure S1).

**Table 1:** Characteristics of the patient.

**Characteristics**	**Patients with PAH (*****n*** **=** **56)**		
	**Baseline**	**Follow-up**	***p* value**
Male/female, *n*/*n*	21/35		
Age, years	62 ± 15		
PAH subtype, *n* (%)			
Idiopathic PAH	55 (98.2)		
PAH with overt features of venous/capillary involvement	1 (1.8)		
WHO FC, *n* (%)			
I		4 (7.1)	<0.001
II	11 (19.6)	16 (28.6)	
III	40 (71.4)	29 (51.8)	
IV	5 (8.9)	7 (12.5)	
BNP (pg/ml)	133 [65–307]*	89 [29–249]*	0.003
Right heart catheterization		†	
Mean pulmonary arterial pressure, mm Hg	42 ± 10	40 ± 10	0.003
Right atrial pressure, mm Hg	8 [6–10]	8 [6–11]	0.127
Pulmonary vascular resistance, Wood Units	7.6 ± 3.1	6.0 ± 3.0	0.066
Cardiac index, l/min/m^2^	2.4 ± 0.5	2.8 ± 0.7	0.147
Pulmonary arterial wedge pressure, mm Hg	11 [8–13]	10 [9–13]	0.547
Pulmonary arterial capacitance, ml/mm Hg	1.5 [1.0–2.0]	1.7 [1.2–2.7]	0.003
Maximal treatment, *n* (%)			
Monotherapy		19 (33.9)	
Dual therapy		25 (44.6)	
Triple therapy		12 (21.4)	

*PH, pulmonary hypertension; FC, functional class; BNP, B-type natriuretic peptide.*

*Values represent mean ± SD, unless otherwise specified.*

*Available in 55 patients.

*Follow-up right heart catheter data were available in 38 patients*.

**Table 2:** Echocardiographic measurements.

	**Patients with PAH (*****n*** **=** **56)**		
	**Baseline**	**Follow-up**	***P*-value**
Right ventricle			
RV end-diastolic area, cm^2^	27 [22–32]	23 [18–29]*	<0.001
RV end-systolic area, cm^2^	20 [16–24]	15 [12–19]*	<0.001
Fractional area change, %	25 ± 11	29 ± 11*	0.010
TAPSE, mm	20 [18–22]	21 [19–23]	0.105
PASP, mmHg	67 ± 23^†^	61 ± 21	0.089
TAPSE/PASP, mm/mmHg	0.29 [0.21–0.40]†	0.35 [0.27–0.44]	0.109
RV global longitudinal strain, %	−15.1 ± 4.7	−16.8 ± 4.8	0.007
Tricuspid valve regurgitation			<0.001
None/mild	23 (41.1)	26 (46.4)	
Moderate	29 (51.8)	21 (37.5)	
Severe	4 (7.1)	9 (16.1)	
Right atrium			
RA area, cm^2^	17 [15–20]	15 [12–20]	0.014
Peak longitudinal strain, %	31 [23–36]	29 [22–39]	0.864
Passive strain, %	8 ± 5	10 ± 8*	0.0117
Peak active contraction strain, %	21 ± 7	20 ± 10*	0.704
Inferior vena cava diameter, mm	18 [15–20]^†^	18 [14–21]^§^	0.928

*PAH, pulmonary arterial hypertension; PASP, pulmonary arterial systolic pressure; RA, right atrial; RV, right ventricular; TAPSE, tricuspid annular plane systolic excursion.*

*Values represent mean ± SD or median (interquartile range) (for normally or non-normally distributed parameters, respectively), unless otherwise specified.*

*Available in 55 patients.

*Available in 52 patients.*

*Available in 54 patients.*

*^§^Available in 49 patients*.

Median (interquartile range) time to echocardiographic follow-up was 11 (9–12) months. The majority of patients (66%) received specific dual or triple combination therapy as maximal treatment. Under specific treatment, pulmonary hemodynamic indices and RV remodeling showed substantial improvement (Tables 1, 2). However, RA PLS, PS, and PACS remained unchanged despite significantly decreased RA size during follow-up.

Intraclass correlation coefficients and coefficients of variation showed good inter- and intraobserver agreement for RA PLS (Supplementary Table S1).

### Clinical Relevance of Longitudinal RA Function

We observed significant associations of baseline RA PLS, PS, and PACS with key baseline parameters (Supplementary Table S2). Among various associations, we observed a strong correlation of baseline RA PLS with baseline RV global longitudinal strain (*rho*: −0.639; *p* < 0.001) and B-type natriuretic peptide (BNP) (*rho*: −0.569; *p* < 0.001).

**Figure 2:**
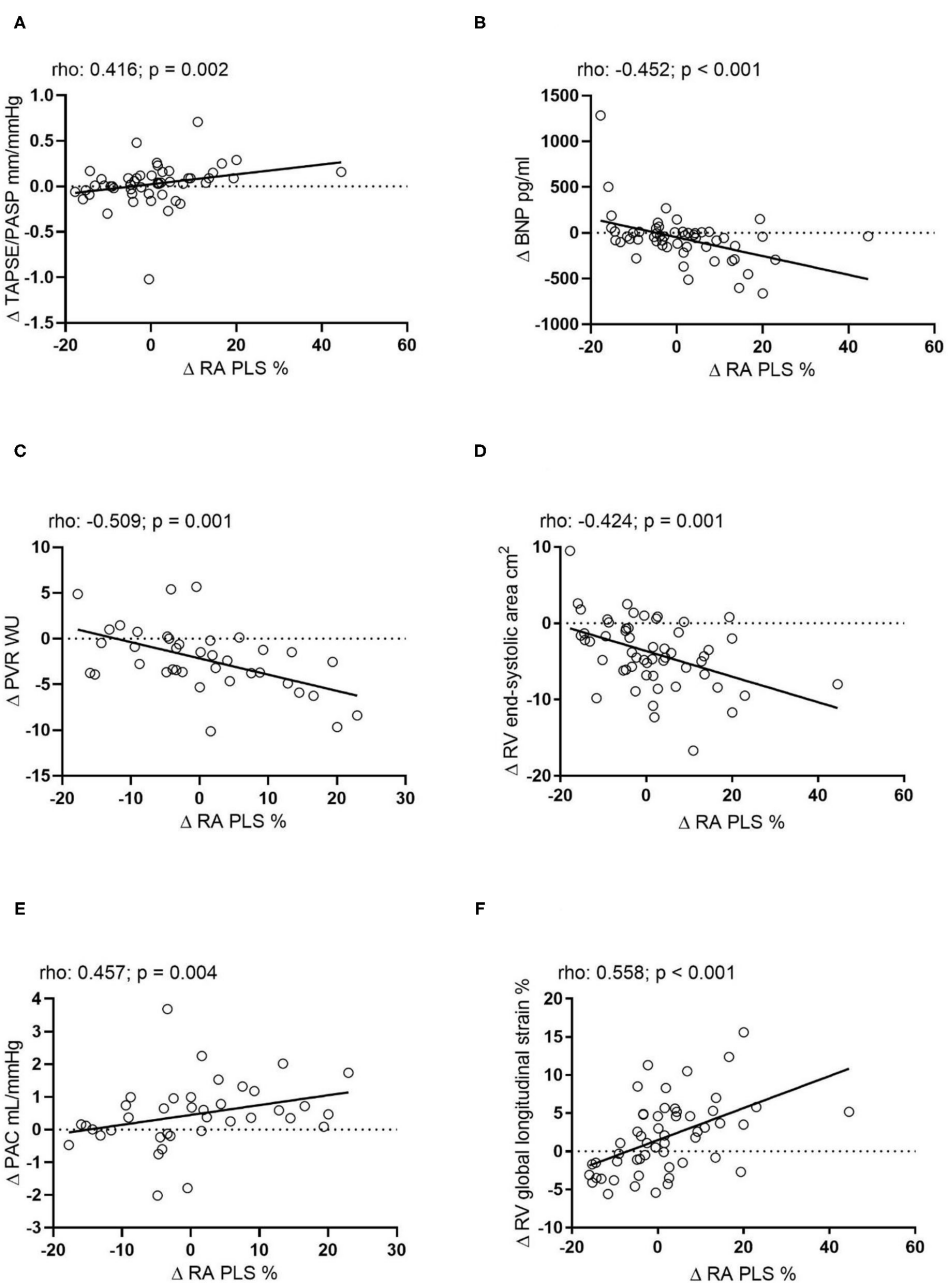
Correlation of the absolute change of RA PLS during echocardiographic follow-up with the absolute change of **(A)** TAPSE/PASP (*n* = 44), **(B)** BNP (*n* = 54), **(C)** PVR (*n* = 38), **(D)** RV end-systolic area (*n* = 55), **(E)** PAC (*n* = 38), and **(F)** RV global longitudinal strain (*n* = 54). Δ, change; BNP, B-type natriuretic peptide; mPAP, mean pulmonary arterial pressure; PAC, pulmonary arterial capacitance; PASP, pulmonary arterial systolic pressure; PVR, pulmonary vascular resistance; RA PLS, right atrial peak longitudinal strain; RV, right ventricular; TAPSE, tricuspid annular plane systolic excursion; WU, Wood Units.

The difference of RA PLS (Δ RA PLS) from baseline to follow-up was significantly associated with a change of the following parameters during follow-up: Δ TAPSE/PASP, Δ BNP, Δ PVR, Δ PAC, Δ mPAP (*rho*: −0.428; *p* = 0.008; plot not shown) and Δ RV end-systolic area. Of note, we observed the strongest association of Δ RA PLS with Δ RV global longitudinal strain (Figure 2). Δ RA PACS was significantly associated with Δ BNP (*rho*: −0.400; *p* = 0.003), Δ PVR (*rho*: −0.341; *p* = 0.036) and Δ PAC (*rho*: −0.349; *p* = 0.032), while Δ PS was only associated with Δ RV end-diastolic area (*rho*: −0.323; *p* = 0.017; plots not shown). Of note, no association was observed between Δ PS and Δ RV global longitudinal strain (*p* = 0.204; plot not shown).

In the multivariate linear regression analysis model 1 (including Δ PVR, Δ PAC, and Δ mPAP), we found that Δ PVR was independently associated with Δ RA PLS [multivariate B-coefficient (95% CI): −1.59 (−2.44 to −0.73); *p* < 0.001]. In the corresponding multivariate model 2 (including Δ TAPSE/PASP, Δ FAC, and Δ RV end-systolic area), we found that Δ RV end-systolic area was independently associated with Δ RA PLS [multivariate B-coefficient (95% CI): −1.09 (−1.74 to −0.45); *p* < 0.001]. Model 3 (including Δ TAPSE/PASP, Δ FAC, and Δ RV global longitudinal strain) showed that Δ RV global longitudinal change was independently associated with Δ RA PLS [multivariate B-coefficient (95% CI): 1.16 (0.60–1.72); *p* < 0.001]. In addition, model 2 showed that Δ TAPSE/PASP was significantly associated with Δ RA PS [multivariate B-coefficient (95% CI): 14.43 (4.82–24.05); *p* < 0.001]. Of note, the models could not identify a significant predictor for Δ RA PACS (data not shown).

**Figure 3:**
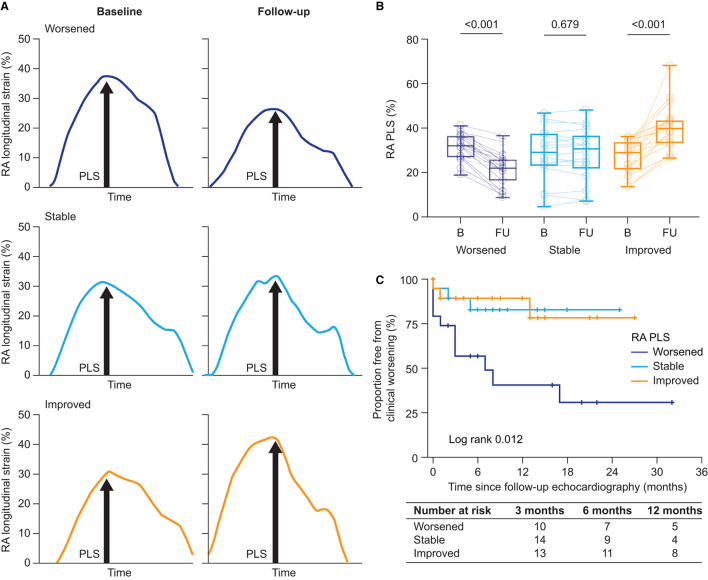
Longitudinal RA function. **(A)** Illustration of the assessment of RA phases at baseline and during follow-up according to the change in RA function (worsened, stable, and improved). Stratification was based on tertile of absolute change of RA PLS and RA PACS. **(B)** RA PLS stratified by tertile of absolute change [tertile I (worsened): Δ −17.8% to −4.2%; tertile II (stable): Δ −4.2% to 4.0%; and tertile III (improved): Δ 4.0% to 44.6%]. Box-plots show median, interquartile range, and minimum to maximum values. **(C)** The Kaplan–Meier plots of time to clinical worsening since follow-up echocardiography stratified by RA function based on RA PLS. RA, right atrial; PACS, peak active contraction strain; PLS, peak longitudinal strain.

For further analysis, the patients were grouped into tertiles according to their Δ RA PLS: worsened (Δ −17.8 to −4.2%), stable (Δ −4.2% to 4.0%), and improved (Δ 4.0% to 44.6%) longitudinal RA function (Figures 3A,B). Δ RA PS and PACS were grouped in an analogous manner (Supplementary Figure S2). As shown in Table 3, no significant differences in baseline hemodynamic or echocardiographic parameters were observed when stratifying by tertile of Δ RA PLS. However, during follow-up, patients with improved RA PLS showed a significantly greater reduction of mPAP and PVR and improvement of PAC, RV strain, TAPSE/PASP, and BNP compared with patients with stable or worsened RA PLS (Table 3). Finally, we grouped Δ RA PLS, Δ RA PS, and Δ RA PACS according to the number of specific vasoactive treatments used (mono, dual, or triple therapy; Supplementary Figure S3). Δ RA PLS and Δ RA PACS showed no significant differences dependent on the treatment regimen used, while Δ RA PS was highest in patients receiving monotherapy. Of note, we observed a reduction of RV end-systolic area in both treatment groups (monotherapy and combination therapy). However, an improvement in FAC was only observed in those patients receiving combination therapy (Supplementary Figure S4).

**Table 3:** Key baseline parameters and changes during follow-up stratified by tertile of longitudinal RA function.

**Characteristics**	**RA peak longitudinal strain tertile**			
	**Worsened (*n* = 19)**	**Stable (*n* = 18)**	**Improved (*n* = 19)**	***p* value**
Baseline				
BNP, pg/ml	106 [88–220]	182 [44–559]*	160 [62–429]	0.797
Mean pulmonary arterial pressure, mm Hg	41 ± 10	40 ± 9	46 ± 11	0.187
Right atrial pressure, mm Hg	8 [5–9]	8 [6–13]	8 [6–10]	0.508
Pulmonary vascular resistance, Wood Units	7 ± 3	7 ± 3	9 ± 3	0.178
Cardiac index, l/min/m^2^	2.4 ± 0.4	2.5 ± 0.6	2.3 ± 0.5	0.399
Pulmonary arterial capacitance, ml/mmHg	1.4 [1.1–2.3]	1.6 [1.1–1.9]	1.4 [0.9–1.9]	0.624
RV end-diastolic area, cm^2^	23 [18–29]	28 [23–35]	28 [25–32]	0.095
RV end-systolic area, cm^2^	18 [12–22]	18 [17–20]	22 [17–27]	0.131
Fractional area change, %	27 ± 8	24 ± 14	24 ± 10	0.655
TAPSE/PASP, mm/mmHg	0.29 [0.22–0.40]	0.31 [0.21–0.46]	0.28 [0.18–0.41]	0.668
RV global longitudinal strain, %	−17.2 ± 3.7	−13.1 ± 5.5	−14.7 ± 4.3	0.050
RA area, cm^2^	17 [14–20]	18 [17–20]	18 [14–22]	0.288
Δ During follow-up				
Δ Mean pulmonary arterial pressure, mm Hg	2 ± 10^†^	−7 ± 11	−8 ± 8^†^	0.039
Δ Pulmonary arterial capacitance, ml/mm Hg	−0.02 [−0.53–0.27]^†^	0.63 [−0.11–1.00]	0.88 [0.36–1.4]^†^	0.006
Δ Pulmonary vascular resistance, Wood Units	−0.1 ± 3.0^†^	−2.4 ± 3.7	−4.2 ± 2.8^†^	0.009
Δ BNP, pg/ml	0 [−71–68]	−61 [−154–6]^§^	−83 [−305 to −21]	0.015
Δ TAPSE/PASP	−0.02 [−0.08–0.02]	0.04 [−0.04–0.14]*	0.09 [0.03–0.17]	0.018
Δ RA area, cm^2^	1 ± 7	−3 ± 4	−3 ± 5	0.083
Δ RV end-diastolic area, cm^2^	−2 ± 6*	−4 ± 3	−5 ± 5	0.103
Δ RV end-systolic area, cm^2^	−1 ± 4	−5 ± 4	−6 ± 5	0.005
Δ Fractional area change, %	1 ± 11^¶^	6 ± 13	7.1 ± 15	0.271
Δ RV global longitudinal strain, %	−1.7 [−3.7 to −0.7]*	1.2 [−0.9–4.8]	4.6 [2.6–5.8]	<0.001

*Δ, change; BNP, B-type natriuretic peptide; PASP, pulmonary arterial systolic pressure; RA, right atrial; RV, right ventricular; TAPSE, tricuspid annular plane systolic excursion.*

*Values represent mean ± SD or median (interquartile range) (for normally or non-normally distributed parameters, respectively), unless otherwise specified.*

*Available in 17 patients.

*Available in 13 patients.*

*Available in 12 patients.*

*^§^Available in 16 patients.*

*^¶^Available in 18 patients*.

### Prognostic Impact of RA Functional Response to Treatment

In total, 17 clinical worsening events [12 hospitalizations (including four escalations of specific PAH therapy and five deaths) were observed during a median follow-up period of 6 (2–14) months (mean 9 ± 8 months) after the echocardiographic follow-up. First, we explored the prognostic relevance of Δ RA function as a continuous variable in the univariate Cox regression analysis. Δ RA PLS (per one unit increase) was significantly associated with the composite endpoint with a hazard ratio of 0.925 (95% CI: 0.873–0.981; *p* = 0.009), while Δ PS (*p* = 0.085) and Δ PACS (*p* = 0.167) were not.

Second, we performed the univariate Cox regression analysis with the RA PLS treatment response patterns stratified by tertile. The pattern was significantly associated with clinical worsening. Patients with worsening of RA PLS during follow-up (tertile I) showed a hazard ratio of 4.16 (95% CI: 1.15–14.96; *p* = 0.029) for the composite endpoint. Similarly, worsening of RA PS during follow-up (tertile I) was significantly associated with the composite endpoint with a hazard ratio of 4.93 (95% CI: 1.08–22.54; *p* = 0.040). Patients with stable RA PS or stable PLS (tertile II) showed non-significantly increased hazard ratios of 2.76 (95% CI: 0.53–14.32; *p* = 0.226) and 1.10 (95% CI: 0.22–5.47; *p* = 0.907), respectively. In the multivariate Cox regression analysis, adjusting for age and sex, worsening of RA PLS during follow-up was significantly associated with clinical deterioration (multivariate hazard ratio: 4.87; 95% CI: 1.26–18.76; *p* = 0.022). This was supported by the Kaplan–Meier analysis which showed a significantly higher clinical worsening event rate in patients with worsened RA PLS compared with patients who had stable or improved RA PLS during follow-up (log-rank *p* = 0.012; Figure 3C). In addition, worsening RA PS remained significantly associated with the composite endpoint within the multivariate model (multivariate hazard ratio: 5.18; 95% CI: 1.13–23.83; *p* = 0.035). However, in the Kaplan–Meier analysis, Δ RA PS stratified by tertile was not able to predict outcome (log-rank *p* = 0.063; plot not shown). Of note, Δ RA PACS was not able to predict outcome in the Cox regression analysis (tertile 1: *p* = 0.319; tertile 2: *p* = 0.972) or the Kaplan–Meier analysis (log-rank *p* = 0.491; plot not shown).

## Discussion

In this study, we have demonstrated that therapy-naïve PAH may show different responses to treatment with respect to RA functional parameters. Moreover, these response patterns are associated with clinically relevant outcome parameters.

Within the last decade, the prognostic and clinical importance of RA function in patients with PH has increasingly come into focus (5, 22). Measurements of altered RA function (reservoir, conduit, or active contractile function) are helpful tools for the evaluation of the severity of RV dysfunction and prognosis in PH (4, 9). Furthermore, RA phasic performance is altered in relation to impaired diastolic function of the chronically overloaded right ventricle, leading to backward venous flow and systemic congestion through RA functional impairment (23). In addition, alterations of RV systolic function accompanied by maladaptive RV remodeling and secondary tricuspid valve regurgitation directly result in loss of phasic RA function, leading to RA remodeling (6, 22). Recently, the longitudinal assessment of RA function after initiation of PAH treatment has been shown to serve as an additional parameter to predict outcome in children with PH (9). It is as of yet unknown whether RA function and its response to PAH treatment during follow-up would also serve as clinically relevant marker in adults.

Δ RA PLS emerged as a clinically relevant parameter in our study. This is consistent with data from Alenezi and coworkers, who identified PLS as the RA parameter of major clinical relevance (4). The relevance of RA PLS may underline the importance of the reservoir function itself and the early impact of RV maladaptation on this specific phase (24). Of note, we observed no prognostic relevance of Δ RA PS (as a measure of the conduit phase) using the Kaplan–Meier analysis, although RA conduit fraction percent (defined as the percentage of total RA area change happening prior to the electrical p wave) was previously associated with risk of adverse events in pediatric PAH (9, 25). Although both parameters mirror RA conduit function, they might not be directly comparable. Furthermore, pediatric PAH might differ significantly from PAH in adults; limited data exist on comparison of these two populations.

Although we observed no general normalization or restoration of RA function in the overall study population after starting PAH treatment, subanalysis revealed different individual patterns of RA functional response by means of changes in PLS. We were able to identify three different patterns of RA function, with either improved, stable, or worsened reservoir function (as mirrored by RA PLS). Moreover, our data indicate that improvement or deterioration of RA function is directly associated with the extent of RV reverse remodeling. Substantial improvement of RA function was associated with a relevant reduction of afterload, pressure, and RV volume under specific therapy. In turn, impairment of RV function during follow-up was directly related to worsening of RA PLS with a subsequent higher probability for a clinical worsening event, highlighting the importance of RA-RV interplay. Improvement of RV function (strain, FAC, and RV volume) after starting PAH treatment was therefore associated with improved RA function. Moreover, our data indicate that RA strain is a dependent variable whose improvements are secondary to improvements in pulmonary arterial and RV parameters. Patients who failed to improve under specific therapy, with no RV reverse remodeling or reduction of afterload, eventually showed worsened RA mechanics as measured by peak RA strain. Of note, RA PLS mirrors RA reservoir function during RV contraction (26). Therefore, the observed association of RV global longitudinal strain with RA PLS indicates that improved RV systolic function also translates into improved RA reservoir function. Again, this highlights the interplay and importance of the RA-RV axis (6).

Decreased afterload leads to better RV function and obviously to better RA filling, presumably through less RV filling at end-diastole and improved venous return. As there is a continuum of elevated PVR, reduced RV function and consecutive impairment of RA function in PH, a failure of the RA-RV axis eventually enhances consecutive dyspnea and congestion (23). Thus, the key target of PH medication is afterload reduction which indirectly improves downstream RV and RA function through improved hemodynamic interplay. Our data emphasize that the RA-RV axis is a unit rather than two separate entities and that RV and RA function are inextricably linked to each other. Therapies directly supporting RA function (27) may play an important role in this context and studies are warranted.

### Limitations

This study has some limitations. First of all, this is a retrospective single-center study and our results may need to be validated in larger prospective cohorts. The sample size and event rate limited the multivariate models and prevented further in-depth analysis. However, to the best of our knowledge, this is the largest study conducted so far focusing on the clinical relevance of RA function in treatment-naïve patients with PAH. Moreover, we were able to provide follow-up data on RA function after treatment initiation, providing additional important information regarding the adaptation of RA function during treatment. The relatively short follow-up period of the study cohort may be an additional limitation.

### Conclusion

After initiation of specific pulmonary vascular therapy, patients with PAH may show different patterns of RA functional response. Recovery of RA functional parameters is significantly related to improvement of RV function. Patients with improvement of RA function in response to PAH therapy have better outcomes than those with stable or worsening RA function. RA functional improvement may thus serve as an additional predictor of treatment response.

## Data Availability Statement

The raw data supporting the conclusions of this article will be made available by the authors, without undue reservation.

## Ethics Statement

All participants gave written informed consent for the enrollment into the University of Giessen PH Registry. The investigation conforms to the Declaration of Helsinki and was approved by the Ethics Committee of the Faculty of Medicine at the University of Giessen (approval #266/11). The patients/participants provided their written informed consent to participate in this study.

## Author Contributions

KT, HAG, WS, HG, and MR contribute to the study design and patient recruitment. KT, HAG, DZ, ZR, WS, HG, and MR contribute to the data collection and analysis. HG and MR contribute to the statistical analyses. KT, HAG, AY, LK, DZ, PD, WS, HG, and MR contribute to the drafting of the manuscript. KT, HAG, AY, DZ, WS, SR, DZ, PD, DB, KO, HG, BB, AY, and MR contribute to the critical revision of the manuscript for important intellectual content. All authors contributed to the article and approved the submitted version.

## Funding

MR and LK received funding from the JLU-CAREER program (German Research Foundation, DFG, 413584448). KT received funding from the Collaborative Research Center (SFB) 1213—Pulmonary Hypertension and Cor Pulmonale, Grant Number SFB1213/1, project B08 (German Research Foundation, Bonn, Germany). PD was funded by the European Respiratory Society—ERS Clinical Training Research Fellowship (CTF202004-00806).

## Conflict of Interest

MR has received support from Bayer; speaker fees from Bayer, Janssen-Cilag GmbH, and OMT and consultancy fees from Bayer and Janssen-Cilag GmbH. PD reports personal fees and non-financial support from Actelion, non-financial support from Astra Zeneca, non-financial support from Bayer, non-financial support from GSK, personal fees and non-financial support from MSD, non-financial support from Novartis, non-financial support from Teva, non-financial support from Boehringer Ingelheim, non-financial support from Vifor, non-financial support from Menarini outside the submitted work. HG has received consultancy fees from Bayer, Actelion, Pfizer, Merck, GSK, and Novartis; fees for participation in advisory boards from Bayer, Pfizer, GSK, Actelion, and Takeda; lecture fees from Bayer HealthCare, GSK, Actelion, and Encysive/Pfizer; industry-sponsored grants from Bayer HealthCare, Aires, Encysive/Pfizer, and Novartis; and sponsored grants from the German Research Foundation, Excellence Cluster Cardiopulmonary Research, and the German Ministry for Education and Research. WS has received speaker/consultancy fees from Abivax, Bayer AG, Liquidia Technologies, Pieris Pharmaceuticals, United Therapeutics and Vectura. HG has received fees from Actelion, AstraZeneca, Bayer, BMS, GSK, Janssen-Cilag, Lilly, MSD, Novartis, OMT, Pfizer, and United Therapeutics. KT has received speaking fees from Actelion and Bayer. The remaining authors declare that the research was conducted in the absence of any commercial or financial relationships that could be construed as a potential conflict of interest.

## Publisher's Note

All claims expressed in this article are solely those of the authors and do not necessarily represent those of their affiliated organizations, or those of the publisher, the editors and the reviewers. Any product that may be evaluated in this article, or claim that may be made by its manufacturer, is not guaranteed or endorsed by the publisher.
