# Supplementary material for: Clinical Relevance of Right Atrial Functional Response to Treatment in Pulmonary Arterial Hypertension
Source: Front Cardiovasc Med. 2021 Dec 7;8:775039. doi: 10.3389/fcvm.2021.775039 (PMC8688770; doi:10.3389/fcvm.2021.775039)
Supplement: Supplementary file 1 [file Data_Sheet_1.docx]

# Supplementary Tables

**Table S1** | Inter- and intra-observer agreement.

|  | **Intraclass correlation coefficient (95% CI)** | | **Coefficient of variation, %** | |
| --- | --- | --- | --- | --- |
|  | **Inter-observer** | **Intra-observer** | **Inter-observer** | **Intra-observer** |
| RA PLS | 0.97 (0.89–0.99) | 0.95 (0.83–0.98) | 5.8 | 6.7 |

*CI, confidence interval; RA PLS, right atrial peak longitudinal strain.*

**Table S2 |** Correlation of RA phasic function with baseline parameters.

| **Parameter** | **PLS** | | **PS** | | **PACS** | |
| --- | --- | --- | --- | --- | --- | --- |
|  | ***Rho*** | ***p* value** | ***Rho*** | ***p* value** | ***Rho*** | ***p* value** |
| **RV global longitudinal strain** | −0.639 | <0.001 | −0.388 | 0.003 | −0.571 | <0.001 |
| **BNP** | −0.569 | <0.001 | −0.528 | <0.001 | −0.347 | 0.009 |
| **mPAP** | −0.350 | 0.008 | −0.299 | 0.025 | −0.155 | 0.254 |
| **RAP** | −0.356 | 0.007 | −0.168 | 0.217 | 0.348 | 0.009 |
| **cardiac index** | 0.337 | 0.011 | 0.180 | 0.184 | 0.342 | 0.010 |
| **PVR** | −0.391 | 0.003 | −0.371 | 0.0035 | −0.204 | 0.131 |
| **PAC** | 0.461 | 0.001 | 0.436 | 0.001 | 0.224 | 0.097 |
| **TAPSE/PASP** | 0.410 | 0.002 | 0.502 | 0.001 | 0.125 | 0.360 |
| **FAC** | 0.423 | <0.001 | 0.488 | 0.001 | 0.218 | 0.107 |
| **RA area** | −0.343 | 0.010 | −0.125 | 0.360 | −0.426 | 0.001 |
| **RV end-systolic area** | −0.482 | <0.001 | −0.341 | 0.010 | −0.445 | 0.001 |

*Spearman’s rank correlation (rho) was used to measure association between variables.*

*BNP, B-type natriuretic peptide; mPAP, mean pulmonary arterial pressure; PAC, pulmonary arterial capacitance; PACS, peak active contraction strain; PASP, pulmonary arterial systolic pressure; PLS, peak longitudinal strain; PS, passive strain; RA, right atrial; RAP, right atrial pressure; RV, right ventricular; TAPSE, tricuspid annular plane systolic excursion.*

# Supplementary Figures


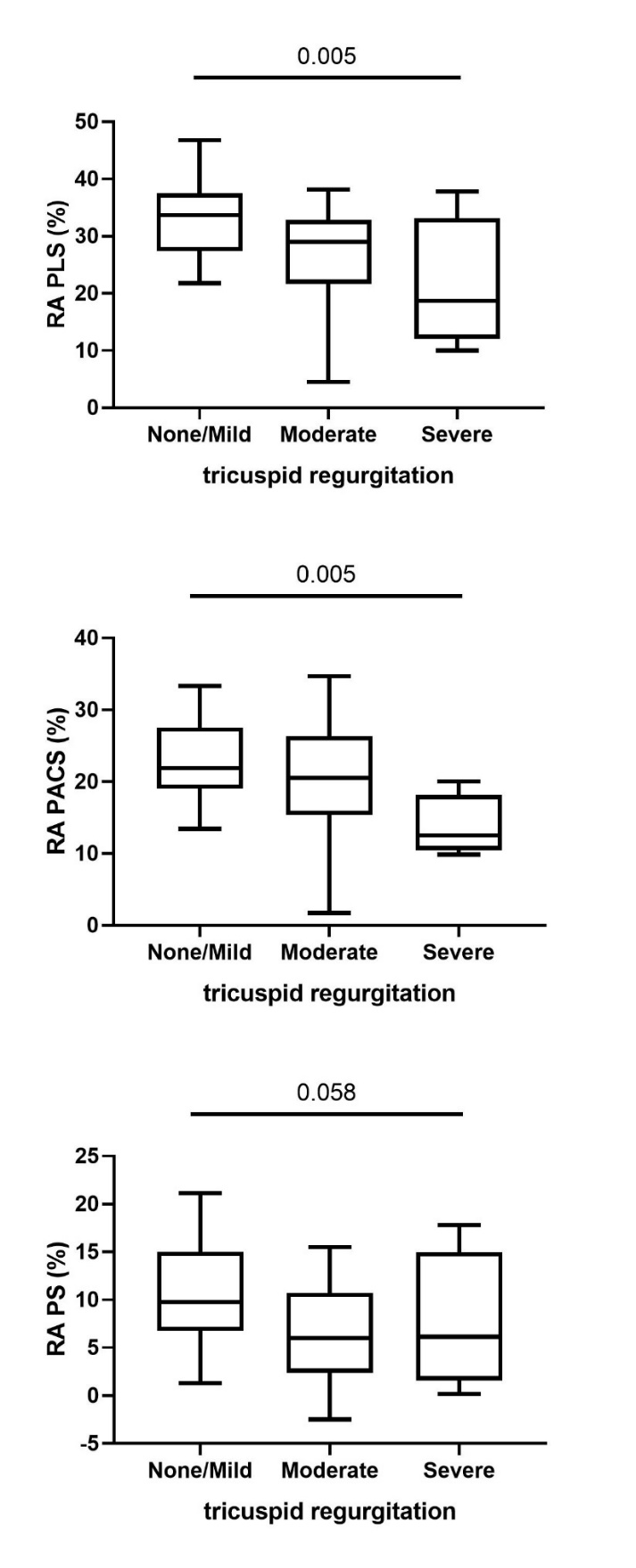


**Figure S1** | Stratification of RA PLS, PS, and PACS according to the severity of tricuspid regurgitation. Box-plots show median, interquartile range, and minimum to maximum values. Independent-samples Kruskal–Wallis test. PACS, peak active contraction strain; PLS, peak longitudinal strain; PS, passive strain; RA, right atrial.

**
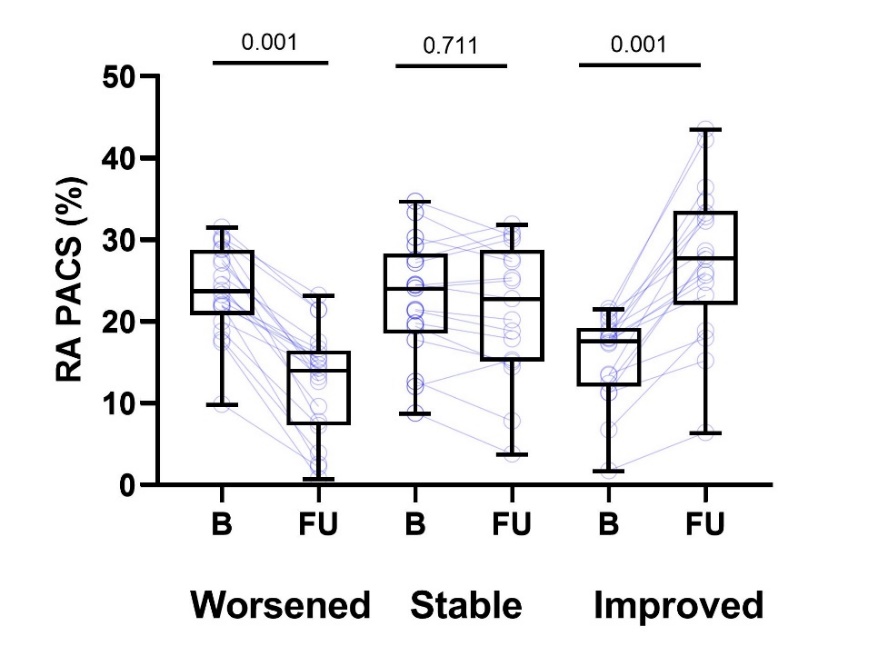
**

**
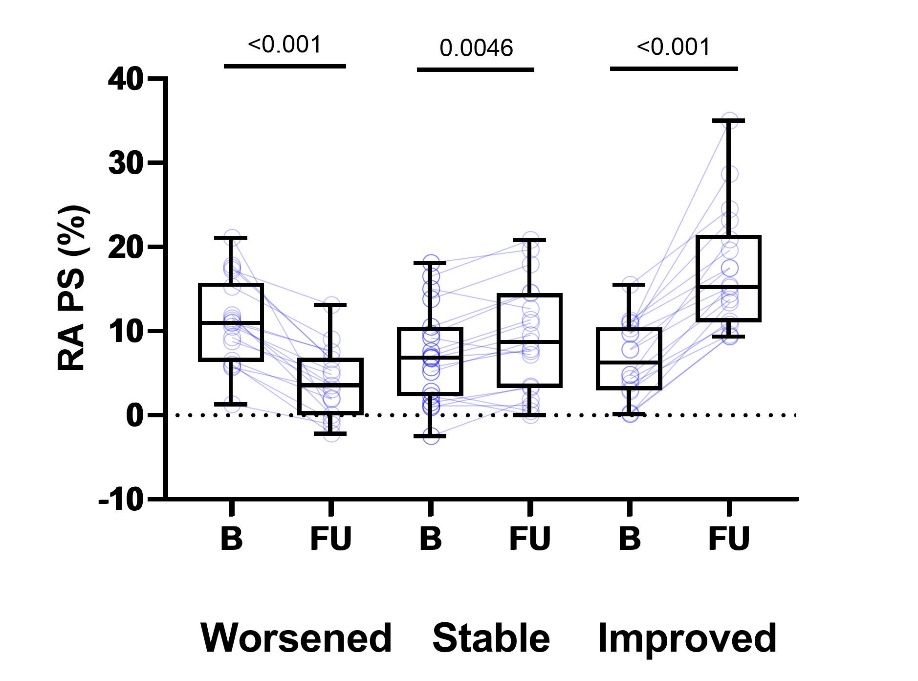
**

**Figure S2** | RA PACS was stratified by tertile of absolute change as shown in the upper graph (tertile I [worsened]: Δ −21.1% to −5.7%; tertile II [stable]: Δ −5.7% to 3.5%; and tertile III [improved]: Δ 3.5% to 22.9%). RA PS was stratified by tertile of absolute change as shown in the lower graph (tertile I [worsened]: Δ −21.1% to −2.4%; tertile II [stable]: Δ −2.4% to 5.0%; and tertile III [improved]: Δ 5.0% to 24.7%). Δ, change; PACS, peak active contraction strain; PS, passive strain; RA, right atrial.

**
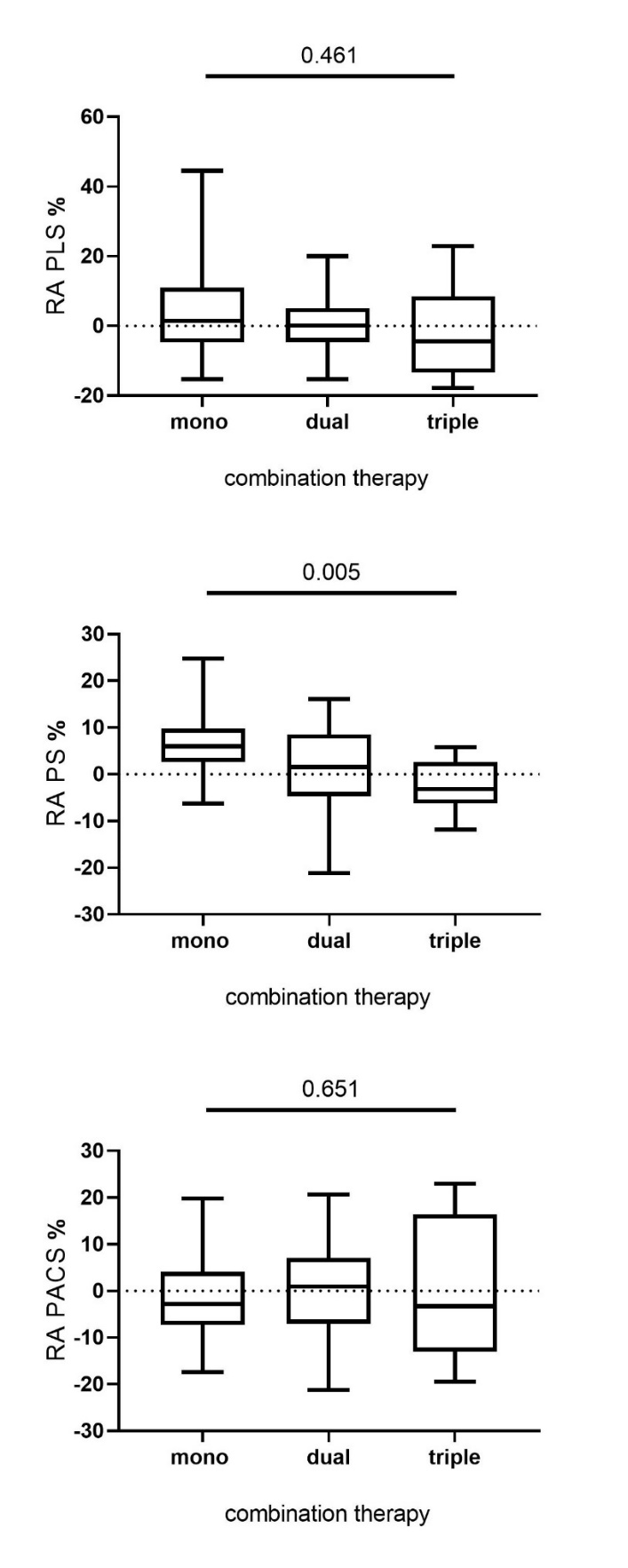
**

**Figure S3** | Stratification of RA PLS, PS, and PACS according to the treatment regimen. Box-plots show median, interquartile range, and minimum to maximum values. One-way analysis of variance. PACS, peak active contraction strain; PLS, peak longitudinal strain; PS, passive strain; RA, right atrial.


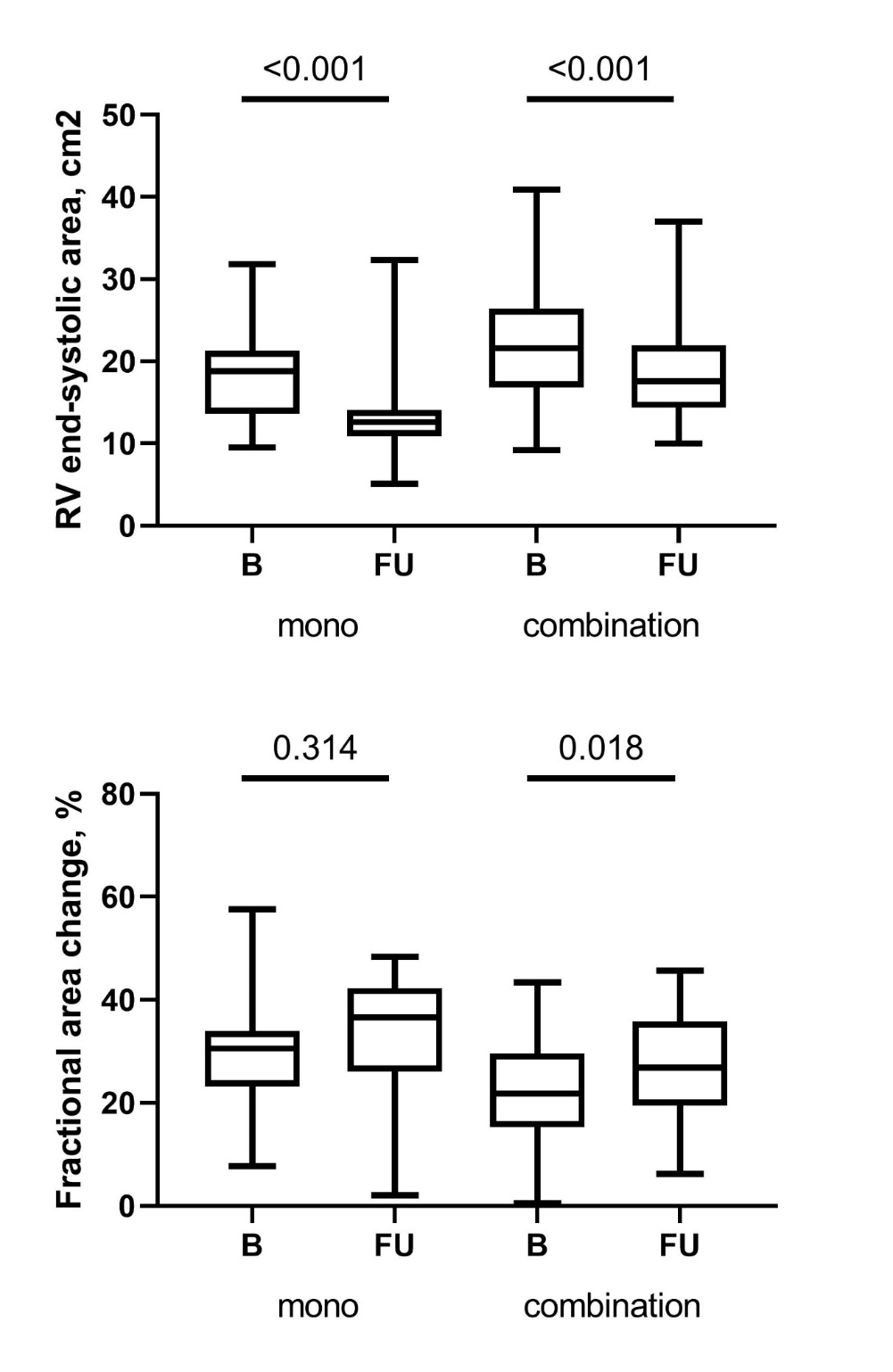


**Figure S4** | Stratification of RV end-systolic area and fractional area change according to the treatment regimen. Box-plots show median, interquartile range, and minimum to maximum values. Related-samples Wilcoxon signed-rank test. B, baseline; FU, follow-up; RV, right ventricular.
